# Variation in cytokine genes can contribute to severity of acetabular osteolysis and risk for revision in patients with ABG 1 total hip arthroplasty: a genetic association study

**DOI:** 10.1186/1471-2350-10-109

**Published:** 2009-10-27

**Authors:** Jiri Gallo, Frantisek Mrazek, Martin Petrek

**Affiliations:** 1 Department of Orthopaedics, Teaching Hospital and Faculty of Medicine and Dentistry, Palacky University, I. P. Pavlova 6, Olomouc 775 20, Czech Republic; 2 Department of Immunology, Teaching Hospital and Faculty of Medicine and Dentistry, Palacky University, I. P. Pavlova 6, Olomouc 775 20, Czech Republic; 3 Laboratory of Immunogenomics, Faculty of Medicine and Dentistry, Palacky University, I. P. Pavlova 6, Olomouc 775 20, Czech Republic

## Abstract

**Background:**

The differences in total hip arthroplasty (THA) survivorship may be influenced by individual susceptibility to periprosthetic osteolysis. This may be driven by functional polymorphisms in the genes for cytokines and cytokine receptors involved in the development of osteolysis in THA, thereby having an effect on the individual's phenotype.

**Methods:**

We performed a study on 22 single-nucleotide polymorphisms (SNPs) for 11 cytokines and two cytokine receptor candidate genes for association with severity of acetabular osteolysis and risk to failure in THA. Samples from 205 unrelated Caucasian patients with cementless type THA (ABG 1) were investigated. Distribution of investigated SNP variants between the groups of mild and severe acetabular osteolysis was determined by univariate and multivariate analysis. Time-dependent output variables were analyzed by the Cox hazards model.

**Results:**

Univariate analysis showed: 1) *TNF*-238*A allele was associated with severe osteolysis (odds ratio, OR = 6.59, *p *= 0.005, population attributable risk, PAR 5.2%); 2) carriers of the *IL6*-174*G allele were 2.5 times more prone to develop severe osteolysis than non-carriers (OR = 2.51, *p *= 0.007, PAR = 31.5%); 3) the carriage of *IL2*-330*G allele was associated with protection from severe osteolysis (OR = 0.55, *p *= 0.043). Based on logistic regression, the alleles *TNF*-238*A and *IL6*-174*G were independent predictors for the development of severe acetabular osteolysis. Carriers of *TNF*-238*A had increased cumulative hazard of THA failure according to Cox model (*p *= 0.024). In contrast, *IL2*-330*G allele predicted lower cumulative hazard of THA failure (*p *= 0.019).

**Conclusion:**

Genetic variants of proinflammatory cytokines TNF-alpha and IL-6 confer susceptibility to severe OL. In this way, presence of the minor *TNF *allele could increase the cumulative risk of THA failure. Conversely, SNP in the *IL2 *gene may protect carriers from the above THA complications.

## Background

Total hip arthroplasty (THA) is a procedure for alleviating end-stage osteoarthritis with significant impact on the quality of life in these patients [1]. However, the life-in-service of joint implants is limited predominantly by aseptic loosening and periprosthetic osteolysis both causally linked to wear debris that are generated continuously by THA [2]. Phagocytosed wear particles provoke macrophages, fibroblasts and other cells to release proinflammatory cytokines and mediators that attract other precursor cells. As a result, chronic inflammatory milieu and a foreign body granuloma develop [3]. This, together with direct inhibition of osteoblast function by wear particles [4], distorts homeostasis in bone in favour of excessive bone resorption, i.e. osteolysis, which can lead to aseptic loosening or periprosthetic fracture.

The concept of cytokines as initiators and perpetuators of particle disease is supported by reports on proinflammatory cytokine (IL-1, IL-6, TNF-α) up-regulation in macrophages and fibroblasts in response to wear particles [5,6]. These pleiotropic cytokines substantially promote the recruitment and maturation of osteoclast precursors at the bone-prosthesis interface [7]. Particle disease/osteolysis might be also facilitated by down-regulation of immunomodulatory cytokines with anti-osteoclastogenic properties (e.g. IL-4, IL-10, IFN-γ) as has been already demonstrated in inflammatory joint conditions [8,9].

However, so called "particle disease" cannot sufficiently explain either the variable degrees of osteolysis found in patients with similar wear rates (and thus exposure to wear particles) or differences in THA survivorship even in the case of the same implant and similar wear rate [10]. For this reason, the concept of individual susceptibility to osteolysis must play a role. Generally, the interaction between the prosthesis and host can be influenced by several less or well known mechanisms including the allergic hypersensitivity, non-allergic and toxic response to a material constituent of the implant [11,12]. Hypothetically, variation in genes for cytokines can alter gene function and/or expression which may affect the individual's resistance/susceptibility to severe osteolysis [13]. In support of this concept, Wilkinson *et al*. reported increased rate of osteolysis in patients carrying the A allele of the *TNF*-238 single nucleotide polymorphism (SNP), [14]. Other studies have addressed variation in single genes for other candidate molecules in relationship with aseptic loosening [15-17]. Surprisingly, only one study reported the variation in the axis of RANKL/RANK/OPG (at position *RANK*+575*T) that is considered the single most influencing regulator of osteoclastogenesis [18]. Interesting results were published by Gordon *et al*. on the association of periprosthetic osteolysis and polymorphism in genes for Wnt canonical pathway (FRZB 200Trp, FRZB 200Arg: 324Arg haplotype), [19]. Recently, the same team identified the association between carriage of *IL1RN *+2018*C allele and a decreased risk of osteolysis after THA [20]. Taken together, none of the reported findings has been replicated in independent samples thus still being considered as preliminary. Therefore, researchers in the field are strongly encouraged to perform well-organized replication studies to enable a meta-analysis [21].

This study was conducted to investigate the contribution of genetic variation in proinflammatory/immunomodulatory cytokine genes to the risk of development of severe periprosthetic osteolysis. In addition, this study addressed the question whether there is an association between particular cytokine gene variants and risk of THA failure.

## Methods

### Subjects

Between February 2004 and June 2007 blood samples were collected by venopuncture from 205 patients with mild and severe acetabular bone defects around THA. The subjects were divided into these two groups according to the size of their acetabular bone defects. This was determined by the classification of Saleh *et al*. from preoperative radiographs and confirmed intraoperatively [22]. Intraoperatively bone defects were evaluated distinguishing at the acetabular site: no significant bone loss (type I), contained bone loss (type II), moderate uncontained bone loss (type III), severe uncontained bone loss (types IV) and pelvic discontinuity (type V). Briefly, if patients fulfilled the criteria for acetabular bone defects of type I and II they were considered as having mild osteolysis (N = 89) while others with more extensive bone defects (types III to V) were classified as severe osteolysis (N = 116).

The study included only Czech Caucasian patients who were operated on at a single institution and those with an identical cementless prosthesis (ABG, Howmedica, Inc., Staines, England). The ABG 1 prosthesis was designed in the 1980s as a press-fit hemispherical cup and anatomical stem both with hydroxyapatite coating. All polyethylene liners were ram-extruded from Hostalen GUR 4150 and air-sterilized with 25 kGy gamma irradiation [23]. Our local register was scrutinized for patients who were and were not revised for osteolysis while the latter were chosen from patients with the longest follow-up assuming those are of "resistant genotype" against premature failure and development of severe osteolysis (phenotypically mild osteolysis). All patients were contacted and invited for clinical and radiographic examination and blood sampling. In patients with bilateral THA who were not revised on either side by the day of blood sampling, the data for hips with longer follow-up were included while in the case of revision, the data for hips with shorter follow-up were recorded. The reasons for that were similar as above, i.e. hypothesized risk genotype for severe acetabular osteolysis development. Basic demographic and clinically-relevant data for both study groups are shown in Table 1.

**Table 1:** Basic characteristics of the THA patients included in the study stratified according to the severity of osteolysis at the acetabular site.

	**Mild osteolysis** **(Types I, II)**	**Severe osteolysis** **(Types III-V)**	**p Value**^#^
Patients, N	89	116	

Gender (men/women)	35/54	33/83	*p *= 0.101

Age at index surgery	48 (27-58)	45 (24-68)	*p *= 0.128

Primary diagnosis:			
Osteoarthritis	35	13	
Dysplastic hip	23	62	***p *< 0.001**
Other diagnoses	31	41	

BMI (kg/m^2^)	28.1 (20.3-35.7)	27.2 (16.0-42.6)	*p *= 0.062

Revision (yes)	44	113	***p *< 0.001**

Age at event* (years)	55 (34-69)	52 (29-77)	***p *= 0.005**

Time to event* (years)	9 (2-13)	6 (3-12)	***p *< 0.001**

Harris hip score	78 (14-96)	65 (28-98)	***p *< 0.001**

Prosthesis stable (yes)	82	86	***p *< 0.001**

Linear wear rate (mm/year)	0.22 (0.04-0.92)	0.34 (0.04-2.52)	***p *= 0.009**

Data presented as median and range (minimum to maximum) in parentheses. ^# ^p values for comparison between the groups of patients with mild/severe osteolysis were calculated by Chi-squared test or Mann-Whitney U test as appropriate. *The "event" was defined as THA revision for revised patients or the final visit for non-revised patients.

All hips included in the study had stable prosthesis at the first year after index surgery. Interpretation of final radiographs consisted of evaluation of implant stability, occurrence and extent of osteolysis. This was performed according to well-known and validated criteria [22,24,25]. In the revised cases (N = 157), the radiographic findings were supplemented with intraoperative findings. In this line, radiographic stability was re-coded (=changed) to loosening in cases where an implant instability was revealed after a weak levering of special tools for the cup/stem removal. Wear measurement was made using a Universal-type measuring microscope in the revised cases. Briefly, the methodology relies on the determination of nine three-dimensional coordinates on the surface of the prosthetic ball fixed inside the retrieved polyethylene cups in both the post-use and manufactured positions. Based on it, the centre of the prosthetic ball at each position and total wear can be calculated using the special computational algorithm. Briefly, previously reported accuracy of the method used for polyethylene wear measurement ranged from 1 to 4 μm and 1 to 9 mm^3 ^for linear and volumetric wear, respectively; reliability of the method was also previously assessed [26,27].

All blood specimens were collected under the same conditions. Written informed consent was obtained from each subject and the study was approved by the Ethics Committee of Palacky University and Teaching Hospital in Olomouc.

### Genotyping of cytokine gene single nucleotide polymorphisms

Twenty-two SNPs distributed in 13 genes encoding cytokines and cytokine receptors (IL-1α, IL-1β, IL-1R, IL-1Ra, IL-4Rα, IL-12, IFN-γ, TGF-β, TNF-α, IL-2, IL-4, IL-6 and IL-10) were investigated (see Additional file 1). Candidate genes were chosen in order to reflect a wide spectrum of cytokines and their receptors which have been shown to play a role in the development of osteolysis. The SNPs were selected for each investigated gene based on their previously reported or anticipated functional relevance for cytokine expression and/or structure (e.g. [28]). The location (function) of investigated SNPs within the genes is listed in Additional file 1. The great majority of chosen SNPs became the most widely studied within particular cytokine genes for their possible association with diseases and they are currently considered as "clinically associated" in public databases .

The technique for genotyping cytokine SNPs has been described elsewhere [29]. Briefly, DNA was extracted from peripheral blood by the standard salting-out procedure [30] and the DNA was handled according to ethical rules. Genotyping was done by polymerase chain reaction with sequence-specific primers (PCR-SSP) using the Heidelberg kit (Cytokine Typing Tray kit, University of Heidelberg, Heidelberg, Germany). The protocol has been described elsewhere . In total, 5000 SNP genotypings were performed. We succeeded in assigning the genotype in more than 98% of genotyped samples/SNPs. The nomenclature for the investigated SNPs was adopted from the manual of the Heidelberg kit (Lot No. CYT11). Despite using the typing kit already validated by us [29] and others [31], a subset of six SNPs in *IL1A*, *IL1B*, *IL6*, and *TNF *genes was tested for genotyping concordance by "in-house" PCR-based methodologies [32,33].

### Study design and statistical analysis

This study investigated eventual associations between severity of acetabular osteolysis in THA (primary outcome) and twenty-two SNPs across eleven genes coding for proinflammatory/immunomodulatory cytokines and also for two cytokine receptor genes, all located across ten chromosomes. Distribution of investigated SNP variants was compared by univariate analysis between the groups of patients with severe (N = 116) and mild (N = 89) osteolysis. Three SNPs emerging from this analysis were tested also for association with secondary outcome of the study, i.e. cumulative hazard of THA failure. The revision of THA was considered as the end point in this analysis although it is known that the decision-making on the timing of revision depends on several factors including those unrelated to the prosthesis failure [34]. Subsequently, a multivariate analysis using logistic regression was performed in order to evaluate interactions of different covariates on the primary outcome. Finally, association of investigated cytokine SNP variants with secondary outcome of the study (cumulative hazard of THA failure) was adjusted for severity of osteolysis.

### Comparison of genotypes and carriage rates

Univariate analysis was performed by comparisons of genotype and phenotype frequencies between subgroups of THA patients using χ^2 ^test; the term 'phenotype frequency' (i.e., carriage rate) gives the number of subjects carrying one (or two) copies of a particular allele on one or both (maternal and paternal) chromosomes. Odds ratios (OR) were calculated for carriers of risk allele compared to non-carriers. Population attributable risk (PAR) was calculated according to the protocol described elsewhere [35]. χ^2 ^goodness-of-fit test which compares observed and expected genotype numbers for each investigated SNP was used to test for deviation of genotype distribution from the Hardy-Weinberg (H-W) equilibrium. *P *value less than 0.05 was considered as significant.

### Cumulative estimates and other statistics

Multivariate analyses using logistic regression were performed by forward stepwise (likelihood ratio) method. Cox regression analysis was used to assess cumulative hazard of revision after THA. The time to the event was determined as the time that elapsed since THA implantation to the date of revision or until the final follow-up, whichever occurred first. Homogeneity of the groups was tested using the Chi-squared and Mann-Whitney U tests. All statistics were calculated with the SPSS 15.0 (SPSS Inc, Chicago, IL, USA).

## Results

### Clinical outcome of patients with THA according to the severity of OL

Patients with severe osteolysis had significantly lower Harris Hip Score at the last follow-up in comparison with those with mild osteolysis (mean 65 *versus *78 points, *p *< 0.001). They were revised in 113/116 (97.4%) cases in comparison with 44/89 (49.4%, *p *< 0.001) of patients with mild osteolysis. THA was classified as "stable" in 74.1% (86/116) and 92.1% (82/89) in patients with severe and mild osteolysis (*p *< 0.001), respectively. Furthermore, patients with severe osteolysis were characterised by shorter time to revision/final checking out (median 6 years) when compared with mild osteolysis group (median 9 years; *p *< 0.001). The reasons for revision were as follows: osteolysis around a stable cup 85/34 (in groups with severe/mild osteolysis), aseptic loosening of the cup 27/7 and periprosthetic fracture 3/1. The most frequent location of acetabular osteolysis was zone II (88%) in revised cases according to DeLee and Charnley classification [36]. In unrevised cases, location of osteolysis was in zone II (42%) followed by zones I (28%) and III (12%).

### The distribution of cytokine/cytokine receptor gene SNPs in THA patients

To determine the distribution of the twenty two cytokine/cytokine receptor SNPs, the group of 205 well-characterised THA patients was genotyped using PCR-SSP technique. Of the 22 investigated SNPs, the distribution of genotypes deviated from the Hardy-Weinberg (H-W) equilibrium for one SNP in the group of THA patients as a whole (*TGFB1 *codon 25: *p *= 0.01).

However, we did not observe any deviation from H-W equilibrium for this SNP in our sample of Czech healthy subjects investigated by the same methodology [29]. Furthermore, the deviation of *TGFB1 *codon 25 SNP could occur due to the random fluctuation when H-W testing for multiple markers was applied. We, therefore, used data on this *TGFB1 *SNP for further analyses with caution. This approach is in compliance with the current recommendations for the reporting of genetic association studies [37].

### Association of investigated cytokine/cytokine receptor gene SNPs with severity of acetabular OL in THA

**Figure 1:**
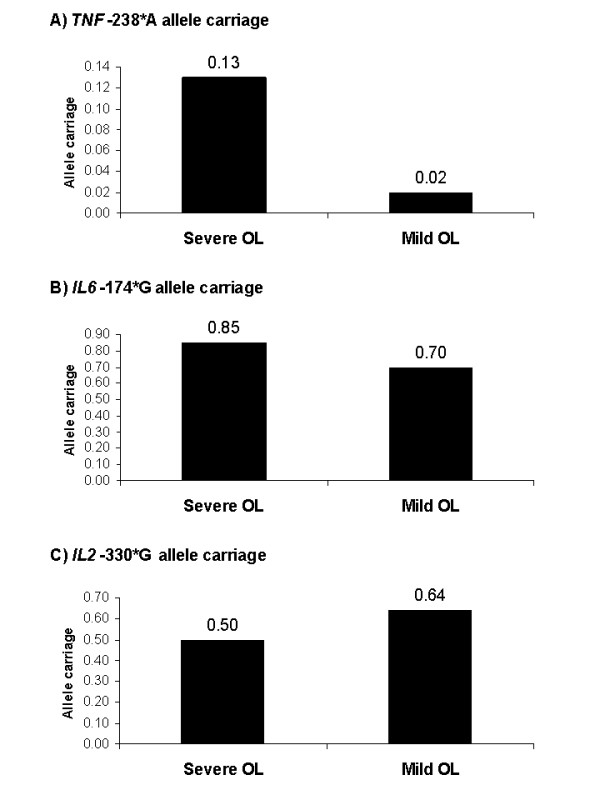
Comparison of proportion of *TNF*-238*A (A), *IL6*-174*G (B), and *IL2*-330*G (C) allele carriers in the groups of patients with severe and mild osteolysis. Comparison severe *versus *mild osteolysis: *TNF*-238*A: *p *= 0.005, OR = 6.59 (95% CI: 1.47-29.64), PAR% = 5.2. *IL6*-174*G: *p *= 0.007, OR = 2.51, (95% CI: 1.27-4.98), PAR% = 31.5. *IL2*-330*G: *p *= 0.043, OR = 0.55, (95% CI: 0.31-0.98). OR: odds ratio, CI: confidence interval, PAR%: population attributable risk percentage

Of 22 analysed cytokine/cytokine receptor SNPs, the proportion of *TNF*-238*A allele carriers was higher among the patients with severe than mild osteolysis (OR = 6.59, *p *= 0.005, PAR = 5.2%, Figure 1A). Similarly, carriers of the *IL6*-174*G allele were overrepresented among patients with severe osteolysis compared to those with mild osteolysis (OR = 2.51, *p *= 0.007, PAR = 31.5%, Figure 1B). By contrast, the *IL2*-330*G allele was underrepresented in patients with severe osteolysis in comparison to those with mild ostelysis (OR = 0.55, *p *= 0.043, Figure 1C) and, thus, appeared to protect against severe osteolysis.

Multivariate analysis of primary outcome (severity of osteolysis) used regression model including age at index THA, gender, weight/height, primary diagnosis, linear wear rate (LWR) and genetic variants (*TNF*-238*A, *IL6*-174*G, *IL2*-330*G). In this analysis, primary diagnosis (*p *= 0.002) and LWR (*p *= 0.004) significantly predicted severe osteolysis. Because LWR covariate strongly limited analysis [it was measured only in revised cases - 157/205 of all THA patients, 76.5%], it was removed from the final regression analysis. In a model without LWR the alleles *TNF*-238*A (*p *= 0.045) and *IL6*-174*G (*p *= 0.049) appeared to be further predictors for the development of more severe acetabular osteolysis, but the contribution of *IL2*-330*G allele was not apparent here.

### Cumulative risk of THA failure and its relationship to the cytokine/cytokine receptor gene SNPs

**Figure 2:**
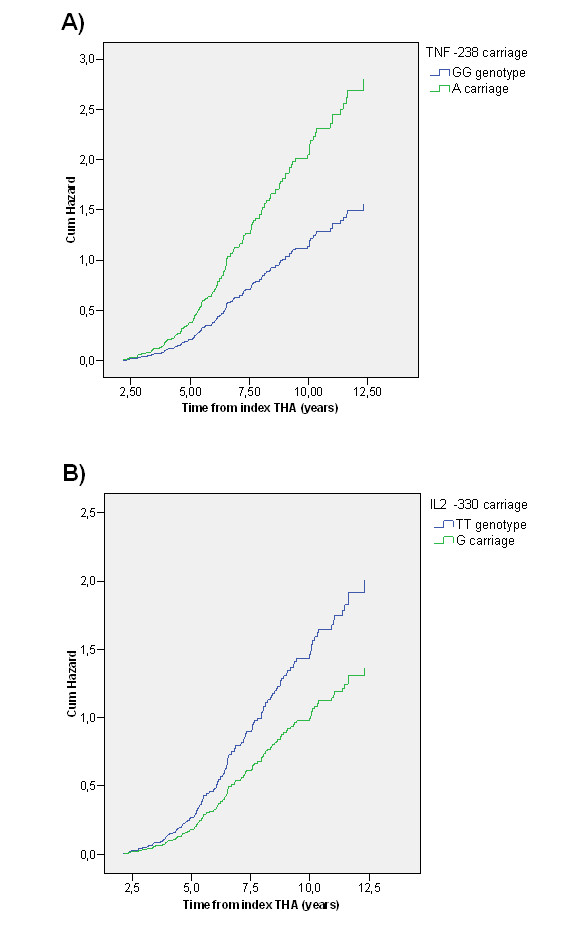
Comparison of cumulative hazard of THA failure between the carriers (green curve) and non-carriers (blue curve) of *TNF*-238*A (A) and *IL2*-330*G (B) alleles assessed by Cox regression analysis. *TNF*-238*A *vs. TNF*-238 GG homozygotes: *p *= 0.024. *IL2*-330*G *vs. IL2*-330 TT homozygotes: *p *= 0.019.

Since the rate of THA revision is considered the most critical parameter in assessing THA outcome and at the same time aseptic loosening together with periprosthetic osteolysis is the most frequent cause of THA failure, we were interested in whether cumulative hazard of THA failure (defined as a revision due to aseptic loosening or osteolysis) was associated with cytokine/cytokine receptor SNP variants. In agreement with the observed associations of the *TNF*-238*A allele with severe osteolysis, *TNF*-238*A carriers were characterised by increased cumulative hazard of THA failure compared to patients with the *TNF*-238 GG genotype (*p *= 0.024, Figure 2A). However, the presence of the *IL6*-174*G allele in THA patients did not change the cumulative hazard of THA failure. On the other hand, the protective effect of the *IL2*-330*G allele was further confirmed in terms of lower cumulative hazard of THA failure in *IL2*-330*G allele carriers compared to non-carriers (*p *= 0.019, Figure 2B). Nevertheless, neither *TNF*-238*A nor *IL2*-330*G alleles predicted changes in the cumulative hazard of THA failure independently from osteolysis as was seen after adjustment of Cox regression analysis for the severity of osteolysis.

## Discussion

This comprehensive study investigated the association between extent of acetabular osteolysis in THA and polymorphic variants across a wide spectrum of genes encoding for cytokines/cytokine receptors with inflammatory and/or immunomodulatory properties. In particular, the study suggests that SNPs in genes for proinflammatory cytokines TNF-α and IL-6 may play a role in the pathogenesis of osteolysis. In addition, a SNP in the gene for the Th1 cytokine IL-2 was found to be a factor associated with severity of acetabular osteolysis and risk for premature THA failure.

In this study, carriage of the *TNF*-238*A allele was associated with severe acetabular osteolysis and risk for premature failure in contrast to the *TNF*-238 "GG" genotype. Individuals carrying the *TNF*-238*A were six times more prone to develop severe osteolysis than patients with the GG genotype. Accordingly, the striking risk for severe acetabular osteolysis associated with *TNF*-238*A carriage could be detected here at statistically significant level though this variant is relatively rare (see power calculations in Additional file 1). Our study, therefore, confirms that *TNF*-238 SNP plays a role in the development of osteolysis as suggested by Wilkinson *et al*. [14]. Concerning another SNP in *TNF *gene promoter, our findings are concordant to those of Wilkinson *et al*. and Kolundzic *et al*. [17], who found no relationship between the carriage of the *TNF*-308*A allele and risk for osteolysis development or premature prosthetic failure.

To properly evaluate the contribution of SNPs in the *TNF *gene to osteolysis development and prognosis of THA, it should be acknowledged that the minor *TNF*-238*A allele occurs rarely in the normal population, limiting its contribution to the overall "population" risk of severe acetabular osteolysis (PAR value, 5.2%). This SNP may, however, serve as a marker for other causative polymorphism(s) in the *TNF *locus or neighbouring polymorphic major histocompatibility complex (MHC) genes. This interpretation would be also in accordance with equivocal data from *in vitro*/*ex vivo *analyses of the functional influence of *TNF*-238 and also *TNF*-308 polymorphisms on *TNF *gene transcription and subsequent cytokine expression, processes which seem to be highly cell or stimulus specific [38,39].

A SNP in the *IL6 *gene promoter was another polymorphism implicated in the pathogenesis of OL in this study. IL-6 is a multifunctional cytokine also involved in the regulation of bone metabolism; therefore its participation in the processes occurring at the bone-prosthesis interface is plausible [6]. Here, *IL6*-174*G allele carriage was associated with the increased risk of severe osteolysis according to both the univariate and multivariate analyses. However, this SNP in gene for IL-6 did not influence the risk for cumulative hazard of THA failure. Association of *IL6*-174*G with severe osteolysis could not currently be supported by straightforward "mechanistic" explanation because its functional role on IL-6 expression is controversial. The allele *IL6*-174*G was originally associated with higher induced expression of IL-6 *in vitro *[40], however this was not subsequently confirmed [41]. Importantly, Terry *et al*. revealed that effect of *IL6 *genetic variation on IL-6 expression is tissue-specific and dependent on other environmental factors [41,42]. This observation may explain the opposite roles of *IL6*-174 SNP observed in different clinical conditions [40,43,44]. Our results, therefore, allow us to speculate that complex local response around THA provides adequate conditions/stimuli promoting "penetrance" of the *IL6*-174 variant in terms of its effect on IL-6 expression. Concerning previous findings on *IL6 *gene in THA outcome, Malik *et al*. [16] like us, reported no association of *IL6*-174 SNP with the risk of premature failure of cemented THA. The association of the *IL6 *SNP (positions -597 and -572) with the earlier prosthetic failure has been reported by Kolundzic *et al*. [17]. However, their study had a small number of patients and included both hips in the same patient into the analysis.

Based on our data the *IL2*-330*G genetic variant may function protectively against severe osteolysis which could result in significantly lower cumulative hazard of THA failure. Interestingly, Campos *et al*. [45] found that polymorphism in the *IL2*-330*G was not associated with premature failure of dental implants. Due to biological and mechanical similarities between THA and dental implants, both studies suggest a protective role of *IL2 *SNP on the prosthetic-bone interface. IL-2 regulates both survival and death of regulatory T cells which might affect the osteoclast life cycle [46]. However, the functional relevance of the investigated *IL2 *SNP is not clear [47,48] and thus the role of IL-2, and therefore also Th1 lymphocytes in the pathogenesis of periprosthetic osteolysis has yet to be elucidated [11]. Whatever the mechanism behind possible IL-2 participation in pathological processes around THA, if proven in other centres/populations, the protective effects of the *IL2*-330*G would have clear implications because the G allele is carried by more than 50% of Europeans [29,49].

While interpreting the results of the current study, one should consider the theory of multifactorial susceptibility to complex disease and current issues of genetic association studies (GAS). Any individual genome contains many functional variants and many of these can influence the development of osteolysis in conjunction with other biological, mechanical and material factors. Furthermore, one may also conceivably argue that other genetic "elements" than those identified in this study may be involved in the complex process of particular disease via phenomenon of linkage disequilibrium [50]. Future investigations should be directed at detailed analyses of specified candidate regions on chromosomes 4q, 6p and 7q in order to search for further genetic markers of osteolysis developing after THA. Finally, based on current experience showing that the great proportion of GAS has not been replicated in independent samples, further validation through replication studies is strictly recommended [51]. In this regard, it should be emphasised that our study replicated the findings of Wilkinson *et al*. for *TNF*-238*A [14].

### Limitations and strengths of our study

We are aware that relative heterogeneity of the primary diagnoses leading to index THA may be linked to the THA outcome including osteolysis. Nevertheless, genetic variants of cytokine genes (namely *TNF*-238*A and *IL6*-174*G) appeared to be independent predictors of severe osteolysis after adjustment for primary diagnosis and other relevant factors in the regression analysis. Moreover, the vast majority of dysplastic hips (over 98%) were of Hartofilakidis type I which influence the THA survival only insignificantly, and the remaining ones were of type II [52]. In addition, selection bias might have an influence on the reported findings because not all patients with the ABG 1 prosthesis could be included in the study (205 of 506). In non-revised cases the severity of bone defects was determined primarily from radiography which could have led to underestimation of the true bone defects. Despite the rigorously created design of this association study, false positive findings cannot be excluded. On the other hand, only patients with identical prosthesis were included which eliminated the influence of inter-prosthetic differences on osteolysis development and prosthetic failure. In addition, surgery was performed at the single institution by a limited number of experienced surgeons which should minimize the role for surgery-related differences on failure of the implant.

Also from the genetic epidemiology view, our study meets the recently adopted criteria for GAS [21]. Apart from emphasis on its preliminary character, this study had adequate study power (more than 80% in 19 of 22 investigated SNPs; see Additional file 1) and adhered to quality control methods in genotyping/analytical methodologies [29]; overall genotyping "failure" rate was very low (less than two percent). Importantly, the parameter of PAR was included to estimate the specific contribution of investigated genetic markers among other complex individual and environmental factors promoting development of severe acetabular osteolysis and premature THA failure.

## Conclusion

This case-control study associated gene polymorphisms in two proinflammatory cytokines TNF-α and IL-6 with extent of acetabular osteolysis. In addition, the risk for THA failure was increased in carriers of *TNF*-238*A allele. In contrast, a polymorphism of the gene for regulatory Th1 cytokine IL-2 emerged as a possible factor protecting against premature failure of THA and was negatively associated with osteolysis severity in univariate analysis. Collectively, the data expand the current paradigm of osteolysis as a predominantly inflammatory process and may implicate T-cells and their modulatory cytokines in the particle disease. If this single-centre data are replicated in other centres/population, new avenues will be opened up for the use of genetic testing in pre-surgical stages and also for investigation of immune response modulation in THA.

## List of abbreviations used in the study

GAS: Genetic Association Study; IFN: Interferon; IL: Interleukins; M-CSF: Macrophage colony stimulating factor; OR: Odds ratio; PAR: Population attributable risk; RANKL: Receptor activator of NF-κβ ligand; SNPs: Single nucleotide polymorphisms; TGF-β: Transforming growth factor beta; THA: Total hip arthroplasty; TNF-α: Tumour necrosis factor alpha.

## Competing interests

The authors declare that they have no competing interests.

## Authors' contributions

All three authors equally contributed to this paper; the order of the authors is alphabetical. JG recruited THA patients into the study and collected clinical details, MP provided healthy population control. FM carried out the genotyping and performed statistical analyses with help of MP's and JG's ideas. Conceptualization and design of the study was joint work of JG (clinical problem of individual predisposition to osteolysis) and MP (immunogenetic approach to its solution and proposal for data analyses/presentation). All three authors contributed to drafting of the paper; the author responsible for the MS integrity is MP.

## Authors' information

All three authors are with the Faculty of Medicine and Dentistry, Palacky University, Czech Republic and in parallel with the Faculty Hospital Olomouc, Czech Republic. Associated Professor Jiri Gallo, MD, PhD is the chief of Department of Orthopaedics; Assistant Professor Frantisek Mrazek, MD, PhD is at the Department of Immunology and leads the DNA section of the Tissue Typing laboratory, Faculty Hospital; Professor Martin Petrek, MD, PhD, is the acting head of the Department of Clinical Chemistry & Immunogenetics, the Principal Investigator at the Laboratory of Immunogenomics & Proteomics and the Director of the Tissue Typing Laboratory.

## Pre-publication history

The pre-publication history for this paper can be accessed here:



## Supplementary Material

Additional file 1 **List of investigated cytokine/cytokine receptor SNPs**. The data provided represent the list of investigated cytokine/cytokine receptor SNPs with their gene location, designation in Cytokine CTS-PCR-SSP Tray Kit (University of Heidelberg), NCBI reference SNP cluster report (refSNP), and function/location of each SNP within the particular gene. Click here for file
