# Supplementary material for: Variation in cytokine genes can contribute to severity of acetabular osteolysis and risk for revision in patients with ABG 1 total hip arthroplasty: a genetic association study
Source: BMC Med Genet. 2009 Oct 27;10:109. doi: 10.1186/1471-2350-10-109 (PMC2774318; doi:10.1186/1471-2350-10-109)
Supplement: Additional file 1 — List of investigated cytokine/cytokine receptor SNPs. The data provided represent the list of investigated cytokine/cytokine receptor SNPs with their gene location, designation in Cytokine CTS-PCR-SSP Tray Kit (University of Heidelberg), NCBI reference SNP cluster report (refSNP), and function/location of each SNP within the particular gene. [file 1471-2350-10-109-S1.DOC]

Additional file 1: List of investigated cytokine/ cytokine receptor SNPs

List of investigated cytokine/ cytokine receptor SNPs with their gene location, designation in Cytokine CTS-PCR-SSP Tray Kit (University of Heidelberg), NCBI reference SNP cluster report (refSNP), and function/location of each SNP within the particular gene. The frequency of less common (minor) allele for each SNP is given for the whole groups of patients with THA (N=205) and the population sample of the Czech healthy subjects (Czech ref., N=150). Statistical power to detect difference in minor allele frequency corresponding to odds ratio (OR) = 2 between the groups of THA patients with severe and mild osteolysis was calculated based on the allele frequency in healthy control group for each investigated SNP according to protocol described elsewhere [1].

| Cytokine / Receptor | Gene location | SNP | Ref.SNP | Function/ location | Allele | Allelic frequency | | HWE^#^ | Statistical power |
| --- | --- | --- | --- | --- | --- | --- | --- | --- | --- |
|  |  | designation |  |  |  |  |  | *p* |  |
|  |  |  |  |  |  | THA | Czech ref.* |  | % |
| IL-1α | 2q | -889 T/C | rs1800587 | 5´UTR^&^ | T | 0.30 | 0.30 | 0.08 | 96 |
| IL-1β | 2q | -511 C/T | rs16944 | promoter | T | 0.30 | 0.33 | 0.52 | 96 |
| IL-1β | 2q | +3962 T/C | rs1143634 | coding / synonymous | T | 0.24 | 0.23 | 0.29 | 94 |
| IL-1R | 2q | pst1 1970 C/T | rs2234650 | distal promoter | T | 0.33 | 0.34 | 0.46 | 96 |
| IL-1RA | 2q | mspa1 11100 T/C | rs315952 | coding / synonymous | C | 0.34 | 0.30 | 1.00 | 96 |
| IL-4Rα | 16p | +1902 G/A | rs1801275 | coding / missense | G | 0.19 | 0.20 | 0.10 | 92 |
| IL-12 | 5q | -1188 A/C | rs3212227 | 3´UTR | C | 0.21 | 0.23 | 0.31 | 94 |
| IFNγ | 12q | +874 A/T | rs2430561 | intron | T | 0.47 | 0.49 | 0.82 | 96 |
| TGFβ | 19q | Codon 10 T/C | rs1800470 | coding / missense | C | 0.42 | 0.47 | 0.60 | 96 |
| TGFβ | 19q | Codon 25 G/C | rs1800471 | coding / missense | C | 0.08 | 0.08 | **0.01** | 70 |
| TNF-α | 6p | -308 G/A | rs1800629 | promoter | A | 0.15 | 0.18 | 0.75 | 90 |
| TNF-α | 6p | -238 G/A | rs361525 | promoter | A | 0.04 | 0.04 | 0.32 | 48 |
| IL-2 | 4q | -330 T/G | rs2069762 | promoter | G | 0.33 | 0.31 | 0.55 | 96 |
| IL-2 | 4q | +166 G/T | rs2069763 | coding / synonymous | T | 0.35 | 0.35 | 0.28 | 96 |
| IL-4 | 5q | -1098 T/G | rs2243248 | promoter | G | 0.06 | 0.06 | 0.35 | 60 |
| IL-4 | 5q | -590 C/T | rs2243250 | promoter | T | 0.20 | 0.16 | 0.16 | 88 |
| IL-4 | 5q | -33 C/T | rs2070874 | 5´UTR | T | 0.19 | 0.17 | 0.23 | 90 |
| IL-6 | 7p | -174 G/C | rs1800795 | promoter | C | 0.44 | 0.42 | 0.14 | 97 |
| IL-6 | 7p | nt 565 G/A | rs1800797 | promoter | A | 0.42 | 0.42 | 0.14 | 97 |
| IL-10 | 1q | -1082 A/G | rs1800896 | promoter | G | 0.45 | 0.47 | 0.61 | 96 |
| IL-10 | 1q | -819 C/T | rs1800871 | promoter | T | 0.24 | 0.23 | 0.72 | 94 |
| IL-10 | 1q | -592 C/A | rs1800872 | promoter | A | 0.24 | 0.22 | 0.99 | 93 |

* The data for the Czech healthy population have been adopted from the article by Kubistova *et al.* [2] after enlargement of the population group up to 150 subjects. The distribution of investigated SNPs in THA patients did not differ from that observed in the healthy control population.

^#^ The *p* values for the χ^2^ goodness-of-fit test used to test for deviation of genotype distribution from the Hardy–Weinberg equilibrium (HWE) in the group of THA patients.

^&^ UTR – untranslated region

References:

1. Lalouel JM, Rohrwasser A: Power and replication in case-control studies. *Am J Hypertens* 2002, 15(2 Pt 1):201-205.
2. Kubistova Z, Mrazek F, Tudos Z, Kriegova E, Ambruzova Z, Mytilineos J, Petrek M: Distribution of 22 cytokine gene polymorphisms in the healthy Czech population. *Int J Immunogenet* 2006, 33(4):261-267.
